# Global 3D rocket launch and re-entry air pollutant and CO_2_ emissions at the onset of the megaconstellation era

**DOI:** 10.1038/s41597-024-03910-z

**Published:** 2024-10-03

**Authors:** Connor R. Barker, Eloise A. Marais, Jonathan C. McDowell

**Affiliations:** 1 Department of Geography, University College London, Gower Street, London, WC1E 6BT UK; 2 Harvard-Smithsonian Center for Astrophysics, 60 Garden Street, Cambridge, MA 02138 USA

**Keywords:** Environmental impact, Climate sciences, Atmospheric chemistry, Climate-change mitigation

## Abstract

Satellite megaconstellation (SMC) missions are spurring rapid growth in rocket launches and anthropogenic re-entries. These events inject pollutants and carbon dioxide (CO_2_) in all atmospheric layers, affecting climate and stratospheric ozone. Quantification of these and other environmental impacts requires reliable inventories of emissions. We present a global, hourly, 3D, multi-year inventory of air pollutant emissions and CO_2_ from rocket launches and object re-entries spanning the inception and growth of SMCs (2020–2022). We use multiple reliable sources to compile information needed to build the inventory and conduct rigorous and innovative cross-checks and validations against launch livestreams and past studies. Our inventory accounts for rocket plume afterburning effects, applies object-specific ablation profiles to re-entering objects, and quantifies unablated mass of objects returning to Earth. We also identify all launches and objects associated with SMC missions, accounting for 37–41% of emissions of black carbon particles, carbon monoxide, and CO_2_ by 2022. The data are provided in formats for ease-of-use in atmospheric chemistry and climate models to inform regulation and space sustainability policies.

## Background & Summary

Rapid deployment of megaconstellations containing hundreds to thousands of satellites has driven a recent surge in rocket launches and accumulation of satellites in low-Earth orbit (LEO)^1,2^. The largest operational satellite megaconstellations (SMCs), Starlink and OneWeb, account for the majority of satellites deployed and orbiting in LEO (https://orbit.ing-now.com). Other proposed SMCs include Yinhe, E-Space, and G60 and more than 60,000 additional SMC satellites are planned for launch by 2040^3,4^. In response, regulatory agencies have proposed or implemented strategies to address risks of debris clutter in space^5,6^, such as reducing the maximum post-mission lifetime of LEO satellites from 25 to 5 years^3^. These regulatory changes mostly affect non-megaconstellation missions, as constellation satellites are designed to have short orbital lifetimes^7^. The already frequent re-entry rates of satellites from megaconstellation missions, intentional design of megaconstellation satellites to undergo complete ablation (burn-up) on re-entry to Earth’s atmosphere (https://www.spacex.com/updates/#sustainability), and an increase in the cadence of rocket launches^2^ will increase the release of potentially harmful chemical byproducts into all layers of the atmosphere.

Recent studies have reported that about 10% of stratospheric aerosol particles already contain elements unique to materials of spent satellites and discarded rocket bodies^8^, and that anthropogenic re-entry byproduct emissions are comparable to natural emissions of these from meteors^9–11^. Re-entry ablation and rocket launches both produce alumina particles (Al_2_O_3_) and gaseous reactive nitrogen (NO_x_). Rocket launches also emit black carbon particles (BC) and gaseous chlorine, water vapour, carbon monoxide, and carbon dioxide (CO_2_). Past modelling studies have identified small, yet notable depletion of stratospheric ozone by Al_2_O_3_, NO_x_, chlorine, and BC^9,12–19^ and atmospheric warming caused by BC absorbing incoming sunlight and by Al_2_O_3_ trapping outgoing longwave radiation^9,16,19–21^. CO_2_ emissions are orders of magnitude less than other industries, but are necessary to compute for tracking carbon neutrality goals^22^.

To further elucidate the atmospheric effects of launch and re-entry emissions of a fast-growing industry, including the evolving influence of satellite megaconstellation missions, contemporary, global, vertically resolved emission inventories are required^23^. So far, global emission inventories have been developed for launches covering 1985–2019^9,24–26^, and re-entries for 2019^9,10^, 2016–2022^27^, and projected out to 2040 based on filings with regulatory bodies^10,27,28^. Emissions have also been calculated for specific scenarios such as space shuttle re-entries^11^ and routine launches of rockets fuelled with a specific propellant: hypergolic^14^, hydrogen^29^, kerosene^19^, solid^13,30^, and liquid methane^21^. All global inventories except the 2021 and projected re-entry inventories^10,27,28^ predate the inception of SMCs (2020), only one of the 2019 re-entry inventories computed NO_x_ emissions^9^, and only two of the launch emission estimates accounted for afterburning effects^24,26^. Afterburning in the lower layers of the atmosphere enhances the combustion efficiency of the rocket plume, altering the mix of byproducts emitted during launch^17^.

Here we address the lack of 3D launch and re-entry emission inventories covering the SMC era by developing a quality checked and validated global, hourly, 3D inventory of air pollutant and CO_2_ emissions from rocket launches and object re-entries for the onset of the satellite megaconstellation era (2020–2022). We use altitude-dependent launch emission indices to account for afterburning, generate datasets of emissions and relevant launch and re-entry activity data (launch and re-entry timing and location, propellant mass and type for each rocket stage, and ablated and non-ablated mass of each re-entering object), and identify data entries associated with megaconstellation missions. We provide our data in the widely used NetCDF format and include accessible variable names and descriptors for ease of use to inform space sustainability initiatives and policies, and to assess risk of unablated materials.

The dataset we compile has 63 Gg of rocket propellant consumed in 2022, mostly in the troposphere and stratosphere, and incorporates 3622 re-entering orbital objects and high-altitude suborbital components of orbital launches totalling 11869 tonnes (~12 Gg) in the period 2020–2022. SMCs are a rapidly increasing fraction of total air pollutant and CO_2_ emissions from space activity (26% in 2020 to 33% in 2022). This fraction is highest for carbon-based emissions (BC, CO, CO_2_) at ~40% in 2022.

## Methods

**Fig. 1:**
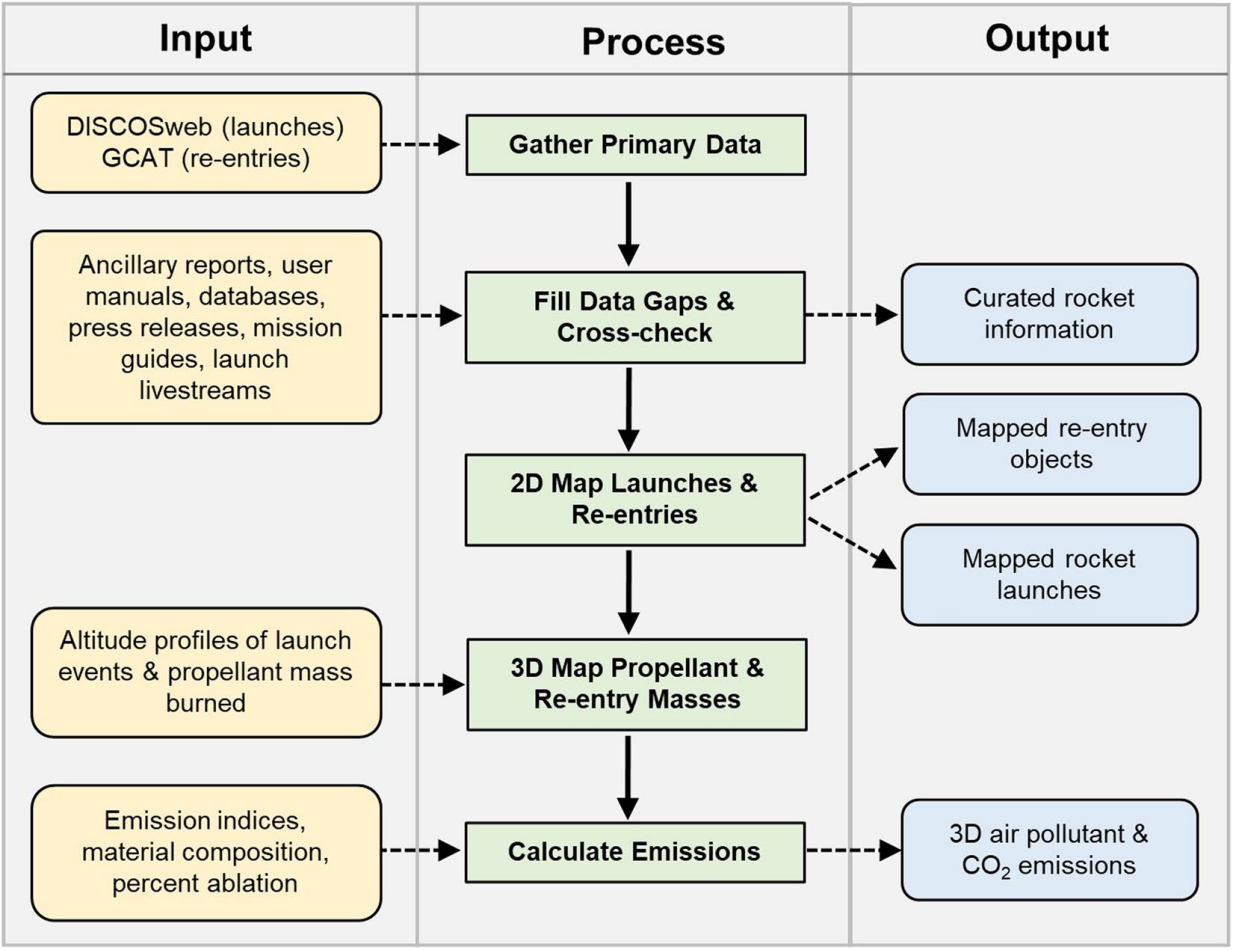
Workflow to generate 3D hourly global air pollutant and CO_2_ emissions from rocket launches and object re-entries for 2020–2022.

Figure 1 summarizes the major steps involved in generating a global, 3D inventory of air pollutant and CO_2_ emissions released up to 80 km altitude (surface to mesosphere) for 2020–2022. Most (~68%) propellant is consumed within this altitude range^20^. The individual processing steps are detailed in the sections that follow.

### Primary data gathering, cross-checks and gap filling

We acquire a complete and cross-checked launch and re-entry activity dataset by consulting data from multiple sources. The primary sources used are the European Space Agency (ESA) DISCOSweb database for launches^2^ and The General Catalog of Artificial Space Objects (GCAT) for re-entries^31^. These are supplemented and cross-checked with routinely updated and archived digital records, mission guides^32–34^, launch vehicle user manuals^35–54^, and launch livestreams. Routinely updated records include Raul’s General SpaceX Map^55^ and Aerospace Corporation CORDS Reentry Database (CRD)^56^. Archived records include Norbert Brugge’s Space Launch Vehicles (SLV)^57^, Spaceflight101 (Sp101)^58^, and Ed Kyle’s Space Launch Report (SLR)^59^. The archived data records end in September 2023 for SLV, March 2022 for SLR, and June 2024 for Sp101.

DISCOSweb^2^ provides the timing, geolocation, launch vehicle details and payload names for orbital rocket launches in 2020–2022. Test and operational launches with SMC payloads are identified as SMC, totalling 86 in 2020-2022 for the Starlink, OneWeb, Yinhe, Lynk, and E-Space constellations. Thirteen of the 86 launches identified as SMCs are rideshares. Of these, 7 are mostly (>95%) SMC payloads, 4 mostly (>90%) non-SMC payloads, and 2 mixed (1 with 25% SMC, the other with 50% SMC payloads). All rockets used to deploy payloads into orbit consist of a series of stages that burn propellant (fuel and oxidiser) to accelerate the rocket toward an orbital velocity^9,14,60^. Fuel types used throughout 2020–2022 include solid, hypergolic, kerosene, and hydrogen. Liquid methane was first used for an orbital launch attempt of a Zhuque-2 rocket in December 2022. The data we collate for each launch includes empty mass, propellant mass, and propellant type for all rocket stages. We also include the mass of the fairings that protect the payload during travel and are discarded as separate halves before payload deployment, typically above 100 km.

The DISCOSweb database^2^ does not provide payload fairing mass and many entries are missing, have outdated data on upgraded rockets, or are inconsistent with other data sources consulted. We address these gaps and cross-check DISCOSweb against multiple ancillary sources, prioritizing launch vehicle information from primary sources such as rocket user manuals and mission guides published by launch vehicle manufacturers. For 57 rockets lacking information from these sources, we use information from Sp101 and SLR. This updates data for 341 of the 446 launches in 2020–2022. We use the average of values reported by these sources for values that are not equal. The difference in values between Sp101 and SLR is typically ≤25%, however there are variations of 27–41% for 5 core stages, and 27–120% for 5 upper stages with relatively small masses (<1 tonne). For 6 rockets (43 launches) missing propellant mass, we obtain propellant masses of individual stages as the difference between the DISCOSweb wet (propellant + rocket body) and dry (rocket body only) masses. We address 36 missing data entries of either propellant mass or stage mass for 24 rockets covering 163 launches using information from the SLV and GCAT databases.

Rocket launch vehicle information is limited to propellant type only for 8 vehicles totalling 25 launches in 2020–2022. These are Astra Rocket 3, Ceres-1, Jielong-3, Kuaizhou-11, Long March 6A (CZ-6A), Long March 11 (CZ-11), Zhongke-1A, and Zhuque-2. We use fairing, stage and propellant mass data for rockets with a comparable length, payload capacity for LEO (destination for most launches in 2020–2022) and, where feasible, propellant type. Proxies with identical propellant types across all stages include Electron for Astra Rocket 3, Shavit for Ceres-1 and Minotaur-1 for CZ-11. Proxies with identical booster and main stage propellant types, but different upper stage propellants (solid for the rockets missing data, hypergolic for the proxies) include Epsilon-2 CPLS for Jielong-3 and Vega-C for Zhongke-1A. The proxy with identical main and upper stage propellant type, but different boosters (solid for the rockets missing data, kerosene for the proxies) is CZ-7A for CZ-6A. Proxies with different propellant types throughout are CZ-6 for Kuaizhou-11 and Antares 230 for Zhuque-2. There are also 7 rockets totalling 23 launches lacking fairing mass data, so we use the average fairing mass reported for all other rockets (~1.8 tonnes). The lack of publicly available rocket propellant and stage mass information, and use of proxies to resolve this, contributes to uncertainties in launch propellant consumption and re-entry mass. Cross-checking and data gap filling increases total launch vehicle dry mass in 2020–2022 from 2.1 Gg in DISCOSweb only to 2.5 Gg and total launch vehicle propellant mass in 2020–2022 from 117.3 Gg in DISCOSweb only to 148.4 Gg. The increases in launch vehicle and propellant mass are primarily due to missing mass data in DISCOSweb.

Altitudes of launch events (engine ignitions and cutoffs) determine the vertical distribution of propellant burned and byproducts released. The launch event altitude data we compile covering the target altitude range (0–80 km) for each rocket includes altitudes of booster engine cutoff (BECO), main engine ignition (MEI), main (first stage) engine cutoff (MECO), and second engine ignition (SEI). Events beyond SEI typically occur far above 80 km (>200 km). Rockets with MEI at altitude are Virgin Orbit’s LauncherOne and the Pegasus XL rocket that are air launched (MEI at ~12 km), and GSLV Mk III that has booster ignition at 0 km and MEI at ~47 km. For all other rockets, MEI is at 0 km. Launch event altitude data are from launch vehicle user manuals, rocket launch mission guides, and rocket launch livestreams. Launch event altitudes for rockets with no publicly accessible information (28 rockets in 2020–2022) are approximated using the average event altitudes of all other rockets with an identical number of stages. For example, the CZ-7A rocket has boosters and three stages, but no publicly available launch event altitude data. We average the altitudes of BECO, MECO, SEI and SECO for all other rockets with boosters and three stages that have accessible information (11 rockets in 2020–2022) to determine the launch event altitude for the CZ-7A rocket. The number of rocket types available to average over ranges from 5 for rockets with 3 stages to 9 for rockets with 4 stages or with boosters and 1–2 stages.

There are 28 launches classified as failed in 2020–2022 in DISCOSweb. We assess the degree of failure using information on the altitude and reason for failure from news reports and media press releases. The launch altitude exceeded 80 km for 21 of the 28 failed launches, so emissions from these launches are included in our inventory. Launch altitude reached ~1–69 km for 6 of the 28, so we include emissions proportional to the altitude reached in the inventory. We assume no propellant consumption and so no emissions for the remaining disputed launch.

**Fig. 2:**
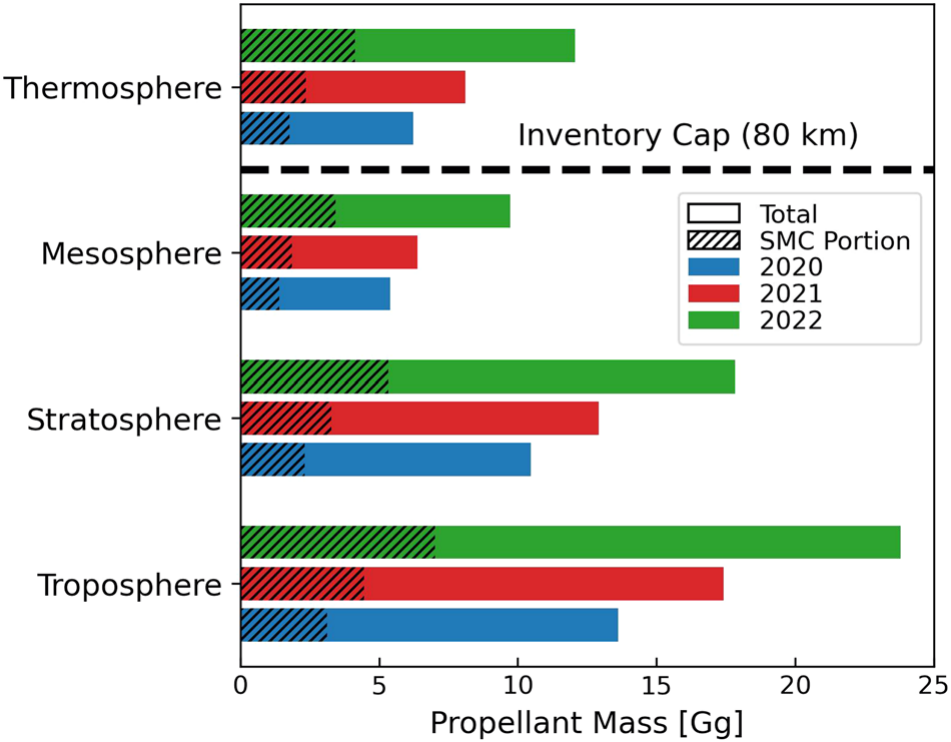
Annual propellant mass consumed by all rockets in each atmospheric layer in 2020–2022. Colours distinguish 2020 (blue), 2021 (red), and 2022 (green). Hatched areas demarcate the SMC portion.

Figure 2 shows the propellant mass consumed in each year and in each atmospheric layer for all launches and for SMC launches only. The number of launches included in the inventory in each year is 114 in 2020, 146 in 2021, and 186 in 2022. The percent contribution of megaconstellation missions to total launches increases from 17% in 2020 to 18% in 2021 and 22% in 2022.

The GCAT database^31^ includes data for orbital and suborbital objects, and rocket parts discarded during launch and payload deployment. The database is split into a series of sub-catalogs. We extract data on the identity, name, category, timing, geolocation, orbital inclination angle, and mass of objects returning to Earth from the sub-catalogs tracking orbital objects (satcat + auxcat), objects below orbit (lcat + rcat), objects returning from other planets (lprcat), objects returning from deep space (deepcat), cargo-crewed capsules (ecat) and objects from failed launches (ftocat). We screen for objects entering at low altitudes and so assumed to experience negligible ablation (apogee < 50 km), as is standard^9,10^. This approach includes slower suborbital stages, introducing a high bias to our re-entry ablated mass. Rocket and mission-specific re-entry velocity data are only reported for 44 of 79 rockets in 2020–2022, so we are not able to screen for slow entering objects with minimal ablation. We apply additional screening to also remove suborbital launches identified with COSPAR IDs of the form YYYY-SNN, military missile tests, and debris fragments from explosions and collisions^31^. The object classes we retain are payloads, rocket stages, and components (functional parts of the spacecraft such as fairings that are designed to be discarded).

Re-entering objects that are not payloads are categorized as SMC if the original launch contained SMC payloads. The original launch is identified using the unique COSPAR launch ID that accompanies each object in the GCAT database. Dry mass data of all objects except cargo mass in our re-entry database are designated as potentially ablatable using dry mass data from GCAT^31^. The ablatable masses of each rocket body object (booster, stage, fairing) from launches in 2020–2022 are set to the dry mass values from our cross-checked launch data. We use the altitude and reason of failure information compiled previously for failed launches to determine re-entry mass from failed rocket stages, unused propellant, and undeployed payloads. We classify failed launch rocket stages and undeployed payloads as ablatable mass and unused propellant as non-ablatable mass.

We scan the CRD^56^ and DISCOSweb database^2^ for objects not included in GCAT^31^. Three object re-entries are added totaling ~2.2 tonnes. Re-entry time is missing for most objects in GCAT. We fill this for 58 objects (32 tonnes) with predicted re-entry times with typical uncertainties of < ± 12 h from objects tracked in the CRD^56^. For the remaining 2,652 objects with no re-entry time data, we use the launch time for the 1,920 objects (8.1 Gg) that re-enter on the same day as the launch and set the re-entry time to 00:00 UTC for the other 732 objects (61 tonnes). The local solar time for these ranges from −12:00 to +12:00 UTC, as the variability in re-entry longitude introduces randomness to the re-entry times.

We cross-check our compiled re-entry data against the launch event altitudes in our launch dataset. Specifically, we check that all boosters and first stages discarded above 50 km during launch that immediately return to Earth are included. This check adds 26 boosters (116 tonnes) and 11 first stages (66 tonnes). We do not check for stages discarded above 100 km, as these objects typically re-enter after months to decades^61,62^ and are anyway included in GCAT. The masses of 124 objects (typically small components) across 2020–2022 are not available from the GCAT, CRD or DISCOSweb databases, and so the ablatable and non-ablatable masses are assigned as NaN.

Re-entering objects in our database total 878 in 2020, 1095 in 2021, and 1649 in 2022. Of these, 218 (2020), 295 (2021), and 447 (2022) are associated with megaconstellation missions. The total mass of all re-entering objects totals 3.2 Gg in 2020 (18% SMCs), 3.8 Gg in 2021 (22% SMC), and 5.0 Gg in 2022 (26% SMC).

### 2D map of global launches and re-entries

We map rocket launches using launch latitudes and longitudes compiled in the previous section corresponding to active spaceports in 2020–2022. We assume that launch emissions are at the same latitude and longitude as the launch site, as rockets typically only deviate horizontally above ~120 km altitude. The reusable first stage of Falcon 9 and the reusable boosters and first stage of the Falcon Heavy rocket burn propellant for controlled return to Earth. The landing zone is typically an autonomous drone ship located ~600 km downrange of the launch site, but some (16 of 112) land at the launch site. We geolocate the emissions from controlled re-entry and landing using landing zone latitudes and longitudes from Raul’s GSM database^55^ for drone ship landings, and DISCOSweb for launch site landings. For drone ship landings missing location data in the GSM database, we use the average drone ship landing position for launches from the same launch site.

**Fig. 3:**
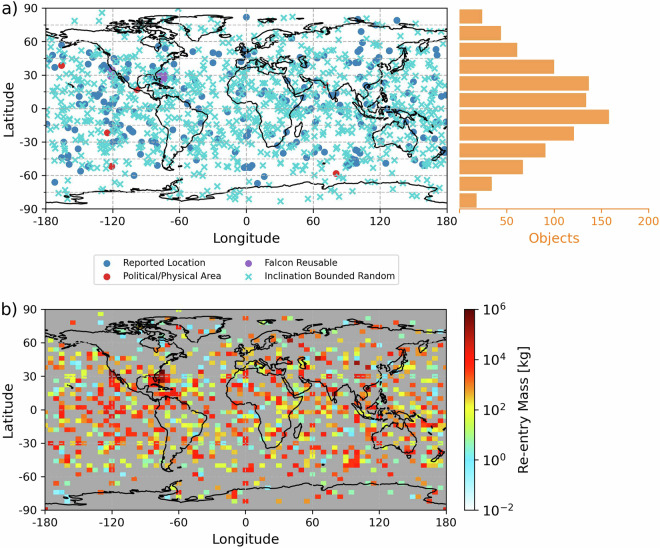
The mapped distribution of re-entering objects (**a**) and mass (**b**) in 2022. In (**a**) the left map is coloured according to the location constraint and the right bar chart sums the number of randomly geolocated objects in 15° latitude bands. The re-entry mass in (**b**) is gridded to a 4° latitude × 5° longitude horizontal grid and is on a log scale. Grey grid cells in (**b**) denote no re-entry mass.

Figure 3 shows mapped re-entry locations and total gridded re-entry mass for objects re-entering in 2022. Object re-entries are mapped using re-entry location information from the GCAT^31^ and GSM^55^ databases. Latitude and longitude information to directly map re-entries is available for 971 objects totaling 4.5 Gg or 38% of total re-entry mass in 2020–2022. For the remaining 62% of re-entry mass, we use other location constraints, where available. We use the same landing data as previously gathered from Raul’s GSM database^55^ to obtain the recovery location of all Falcon rocket reusable stages and Falcon fairings (322 objects, 3.1 Gg), accounting for 29% of total fairing mass in 2020–2022. The re-entry location of 49 objects (0.2 Gg) are listed in the GCAT^31^ as political (country, administrative state) or physical (ocean, continent) areas. For these, the latitude and longitude of re-entry are randomly sampled from a uniform distribution within that given area, with the latitude bounded by the object’s orbital inclination. For example, an object with an orbital inclination of 30° will be geolocated with a latitude between 30°S-30°N. If the orbital inclination of an object is missing in the GCAT^31^, the inclination is set using the largest orbital inclination of all other objects from the same launch.

For 2156 objects (4.1 Gg) with no geographic location in either the GCAT or DISCOSweb databases, only the latitude is bounded by the orbital inclination. This includes non-Falcon fairings. We identify fairing halves from the same launch and assign these the same re-entry latitude and longitude. Objects re-entering attached to a larger object are set to re-enter at the same location as the parent object. The number of randomly geolocated objects re-entering in each 15° latitude band is shown in Fig. 3a. Most re-entry events occur close to the Equator due to the inclination-bounding of latitudes. This pattern is expected from the oblateness of Earth^63^. The re-entry location is uniform across all longitudes.

### 3D global maps of propellant consumption and re-entry mass influx

To determine the vertical distribution of launch emissions, we use the propellant consumption vertical profile of Ross and Sheaffer^20^ that documents the proportion of propellant burned in 5-km altitude bins up to 100 km. We use the BECO, MECO, and SEI altitudes compiled previously to determine the altitude range that individual rocket stages burn propellant. For ease of use in chemical transport models, the propellant consumed by individual rocket launches is interpolated onto vertical grids of 47 and 72 layers over an atmospheric chemically relevant range (≥0.01 hPa, ≤80 km). This maps to the widely used GEOS-Chem atmospheric chemistry transport model grids (https://geoschem.github.io/), or similar models that interpolate emissions to the model resolution. The temporally and spatially varying pressure of each vertical layer is calculated using atmospheric pressure from the offline NASA Modern-Era Retrospective analysis for Research and Applications version 2 (MERRA-2) meteorology that drives the GEOS-Chem model.

We assume that all propellant is consumed between ignition and cutoff, except Falcon series reusable stages that reserve propellant for propulsive landing manoeuvres. Falcon propulsive landing always includes a re-entry burn across 70–55 km^64^ to decelerate the stage and limit damage to the vehicle during atmospheric re-entry^65^ and a landing burn initiated at 3.3 km to land the stage^64^. We set the propellant reserved for re-entry burn as 5.6% of the reusable stage propellant mass and controlled landing burn as 1.2%, following Kim *et al*.^64^. A boostback burn is used for spaceport landings to guide the rocket towards the launch site. We assume this occurs above the altitude limit of our inventory, as the boostback burn occurs shortly after SEI at ~78 km when the first reusable stage is on an upward trajectory. We assume that 5.6% of total first stage propellant is used for this boostback burn^64^. The total propellant used for the Falcon main ascent is then 93.2% of the total for oceanic landings and 87.6% for ground landings. The Electron rocket first stage was not fully reusable for the period under study, although parachute recovery of the stage is being developed. The first stage was recovered for 6 of 22 Electron launches in 2020–2022. All individual object re-entry masses are distributed evenly across the upper to middle mesosphere (0.01–0.21 hPa, 80-60 km).

### Air pollutant and CO_2_ emissions calculation

Propellant dependent launch emissions are calculated for the 7 dominant pollutants and CO_2_. The pollutants include particle-phase BC and Al_2_O_3_, and gas-phase NO_x_, water vapour (H_2_O), carbon monoxide (CO), and chlorine compounds (Cl_y_). H_2_O is common to all rocket propellants. Emissions specific to individual propellants include NO_x_ for nitrogen-containing hypergolic propellant, BC, CO, and CO_2_ for carbon-containing propellants (all except hydrogen fuel), and Cl_y_ and Al_2_O_3_ for solid rocket propellants only. NO_x_ also forms from reaction of atmospheric diatomic nitrogen (N_2_) and oxygen (O_2_) in the hot rocket exhaust plume, so is ubiquitous to all fuel types^66,67^.

Table 1 lists primary emission indices (pEI), the mass of emissions per mass of propellant burned. These are obtained from averaging literature reported values and represent stoichiometric emissions from direct combustion of rocket propellants before accounting for afterburning effects. The largest primary emission sources are hydrogen fuel for H_2_O and kerosene for CO, CO_2_ and BC. Carbon emissions (CO, CO_2_, and BC) from solid fuels result from the hydrocarbon binder that combines the fuel and oxidiser. According to the range in pEIs for compounds and propellant types with multiple estimates, the relative standard deviations suggest uncertainties ranging from 1% to 100%.

**Table 1:** Rocket launch primary emission indices for each propellant type^a^.

Propellant	Primary Emission Indices (pEI) [g kg^−1^]							
	H_2_O	H_2_^b^	CO	CO_2_^c^	BC	Fuel NO_x_	Al_2_O_3_	Cl_y_
Solid	302^d-g^	25^f^	227^f^	112^d,f^	16^d,f,h^	—	328^d-g^	217^e-g^
Hypergolic	392^d-g,i^	4^f^	69^f^	150^d^	16^d,f,h^	10^f,i^	—	—
Kerosene	340^d-g^	10^f^	318^f^	637^d-f^	22^d,f,h^	—	—	—
Methane	446^f^	6^f^	120^f^	426^f^	5^f^	—	—	—
Hydrogen	1058^d-f,j^	35^f^	—	—	—	—	—	—

^a^Mean of multiple literature sources. ^b^Used to calculate final EIs of H_2_O. ^c^Used in Eq. (1) to calculate final EIs of CO. ^d^Ross *et al*.^20^. ^e^Pradon *et al*.^25^. ^f^CSVEM^67^. ^g^Desain *et al*.^24^. ^h^Maloney *et al*.^83^. ^i^Ross *et al*.^14^. ^j^Larson *et al*.^29^.

Emissions of fuel NO_x_ for hypergolic fuel and Al_2_O_3_ for solid rockets are not affected by afterburning, so values in Table 1 are used directly. For H_2_O, CO, CO_2_, indirect NO_x_, and BC, equations from the Commercial Space Vehicle Emissions Modeling (CSVEM) report^67^ are used to account for afterburning. The CSVEM equations are best fits derived from a limited number of modelling studies for H_2_O, CO, CO_2_, and indirect NO_x_ and modelling studies constrained by ambient and laboratory measurements for BC. Afterburning leads to further combustion of CO to CO_2_, BC to CO_2_, and H_2_ to H_2_O in the hot oxidising rocket plume^17^. As afterburning depends on the availability of atmospheric oxygen, its efficacy decreases with altitude to become negligible for most pollutants in the upper stratosphere (>40 km)^17,20^. H_2_ is assumed to all convert to H_2_O in the rocket plume throughout the launch process, so the final EI for H_2_O is the sum of the pEIs for H_2_O and the H_2_ (Table 1) scaled by the molecular weight ratio of H_2_O and H_2_. Altitude-dependent CO EIs (${{EI}}_{{CO}}$) are calculated as:

1 $${{EI}}_{{CO}}=\min \left[{{pEI}}_{{CO}},0.0025{e}^{0.067\times h}\times \left({{pEI}}_{{CO}}+{{pEI}}_{{{CO}}_{2}}\right)\right]$$

where *h* is altitude in km. ${{EI}}_{{{CO}}_{2}}$ is calculated to maintain carbon molar balance of the sum of the CO and CO_2_ pEIs in Table 1 at each altitude. The altitude-dependent EIs for BC (${{EI}}_{{BC}}$) are calculated as:

2 $${{EI}}_{{BC}}={{pEI}}_{{BC}}\times \max [0.04,\min [1,0.04{e}^{\left(\frac{0.12}{h-15}\right)}]]$$

Symbols are consistent with those in Eq. (1). The formulations in Eqs. (1, 2) cause CO and BC emissions to increase and CO_2_ emissions to decrease with altitude. ${{EI}}_{{CO}}$ increases exponentially from <3 g kg^−1^ at 0 km to pEI_CO_ (Table 1) at 67–84 km, depending on propellant type. ${{EI}}_{{{CO}}_{2}}$ decreases exponentially from a propellant-dependent range of 258–1133 g kg^−1^ at 0 km to ${{pEI}}_{{{CO}}_{2}}$(Table 1) at 67–84 km. ${{EI}}_{{BC}}$ increases exponentially from <1 g kg^−1^ at 0 km to ${{pEI}}_{{BC}}$ (Table 1) at ~42 km. ${{EI}}_{N{O}_{x}}$ emitted as NO from reaction of N_2_ and O_2_ declines exponentially with altitude from 33 g kg^−1^ at 0 km to <1 g kg^−1^ above 14 km:

3 $${{EI}}_{N{O}_{x}}=33\times {e}^{-0.26h}$$

Equations (1–3) are based on just 4, 4, and 11 experimental data points, respectively, at altitudes ≤40 km and are unconstrained above 40 km. The measurements used to derive the equations are also for few types of propellants: solid for CO, kerosene for BC, and solid, kerosene and hydrogen for NO_x_. Given this, the CSVEM equations are likely a large contributor to uncertainties in the magnitude and vertical distribution of rocket launch emissions of CO, CO_2,_ BC, and NO_x_, especially in the upper stratosphere and mesosphere.

**Fig. 4:**
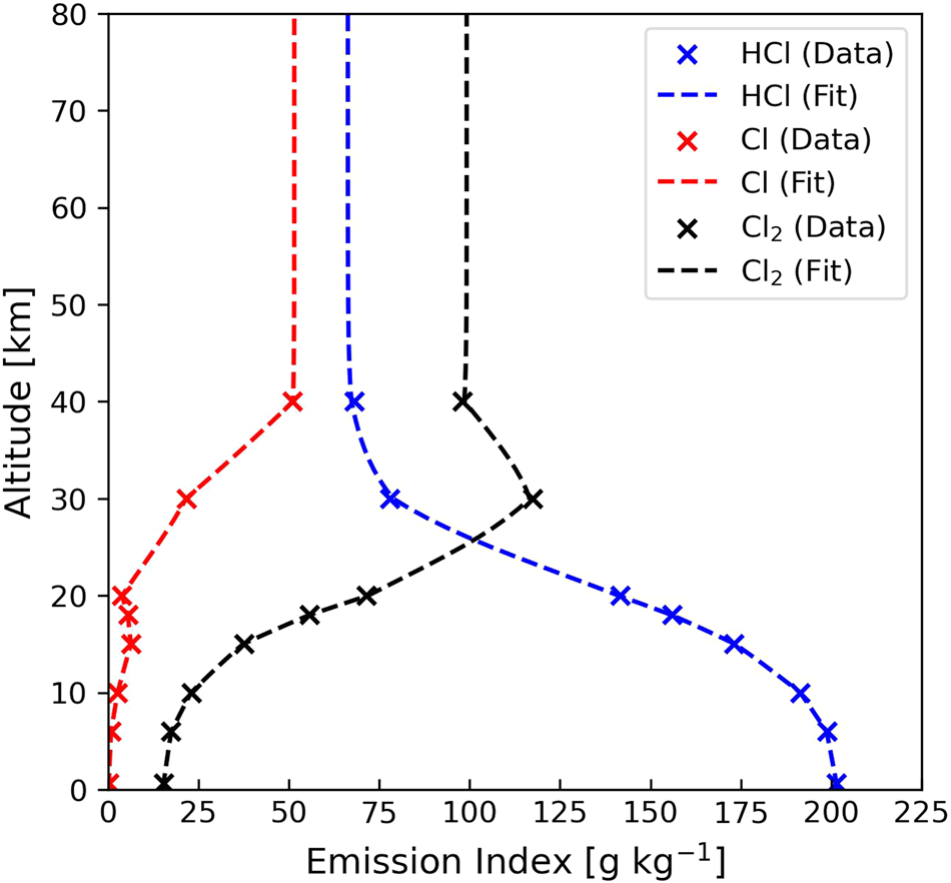
Vertical profiles of Cl_y_ emission indices from 0 to 80 km. Symbols are averaged data from studies of chlorine mass partitioning^68–71^ and the dashed lines are fits to the data for HCl (blue, Eq. (4)), Cl (red), and Cl_2_ (black).

Chlorine (Cl_y_) partitions as HCl, Cl and Cl_2_. This partitioning depends on altitude, due to variability in environmental conditions. The exact partitioning is uncertain, as the chemistry is complex, data are scarce, and literature values are inconsistent^67^. The vertical profiles of HCl, Cl and Cl_2_, obtained as the mean of modelling studies of chlorine mass partitioning^68–71^, are shown in Fig. 4. HCl accounts for the majority of Cl_y_ at 0 km and decreases exponentially with altitude. The fit we derive for HCl is:

4 $${{EI}}_{{HCl}}={{pEI}}_{{{Cl}}_{y}}\times \left[\frac{0.627}{1+{e}^{0.226\left(h-20.9\right)}}+0.304\right]$$

The altitude-dependent shapes of Cl_2_ and Cl deviate from exponential at 30–40 km, so instead we preserve the altitude-dependent shape of the literature means shown in Fig. 4, ensuring chlorine molar balance is maintained. We extend the values at 40 km in Fig. 4 to 80 km.

All altitude-dependent EIs for each propellant type are multiplied by the rocket propellant mass vertical profiles generated in the previous section to calculate vertical profiles of air pollutants and CO_2_ emitted during each launch.

Objects re-entering the Earth’s atmosphere from LEO either partially or fully ablate in the dense layers of the upper atmosphere^72,73^. This high-temperature process produces NO_x_ from heating atmospheric N_2_^11,29^ for all re-entering objects. For those that ablate, Al_2_O_3_ is the dominant metal oxide produced via oxidation of re-entering objects that are mostly comprised of aluminium^10^. Many other gas- and aerosol-phase products would also form^73^, but knowledge and quantification of these is lacking. The ablation chemistry yielding chemical byproducts is complex and depends on many factors, such as the altitude, speed, and angle of entry, and the material composition and structural design of the re-entering object^73,74^. Altitude, speed and angle of entry information are not available for each object and material composition and design of payloads is often proprietary. Instead, we classify objects into broad categories and assign a representative degree of ablation to each. The object classes include discarded core (boosters and first stages) and upper (stages 2–4) stage rocket bodies, discarded fairings, SMC and non-SMC payloads (satellites) and non-fairing components. The proportion ablated we assign to each is 30% for core stages and fairings^10^, 65% for upper stages^10^, 80% for non-SMC payloads and non-fairing components and 100% for SMC payloads and non-fairing components^10^. We assume reusable objects that include crewed and cargo capsules, all Falcon 9 first stages, the 6 recovered Electron first stages, Falcon Heavy boosters and first stages, and Falcon fairings do not ablate at all. This is assumed due to lack of data and because reusable heat shields are typically ablation-resistant ceramic material^72^. Our assumed portion of re-entering mass ablated suggests that over 2020–2022, ~5 Gg of a total of ~12 Gg of re-entering objects return to Earth unablated, increasing from ~1.5 Gg in 2020 to ~2.0 Gg in 2022.

To calculate Al_2_O_3_ emissions, we assume that all rocket body core stages and fairings are 70 (mass) % aluminium^28,75^ and that all payloads and components are 40% aluminium^10^. We also assume that all ablated aluminium converts to Al_2_O_3_, thus yielding an upper estimate of re-entry Al_2_O_3_ emissions. We calculate Al_2_O_3_ emissions for each object as the product of the re-entry mass from our compiled and cross-checked data, the percent mass ablated, and the percent mass that is aluminium. The lack of object-specific data for the percent mass ablated and percent aluminium mass hinders quantification of uncertainties in our re-entry Al_2_O_3_ mass. To calculate re-entry NO_x_ emissions, we assume that the amount of NO_x_ emitted as NO is proportional to the total mass of the object re-entering, as is standard^9^. The percent mass values we use are 17.5% (17.5 kg NO_x_ as NO for a 100 kg object) for reusable objects from a theoretical estimate for space shuttle re-entry^11^, and 40% for discarded objects from a modelling study of spacecraft and upper stage re-entries^72^. Falcon 9 reusable stages re-enter the atmosphere at a much slower velocity (~2 km s^−1^)^64^ than the space shuttle (~7 km s^−1^)^11^, so the 17.5% conversion factor is likely an overestimate.

**Fig. 5:**
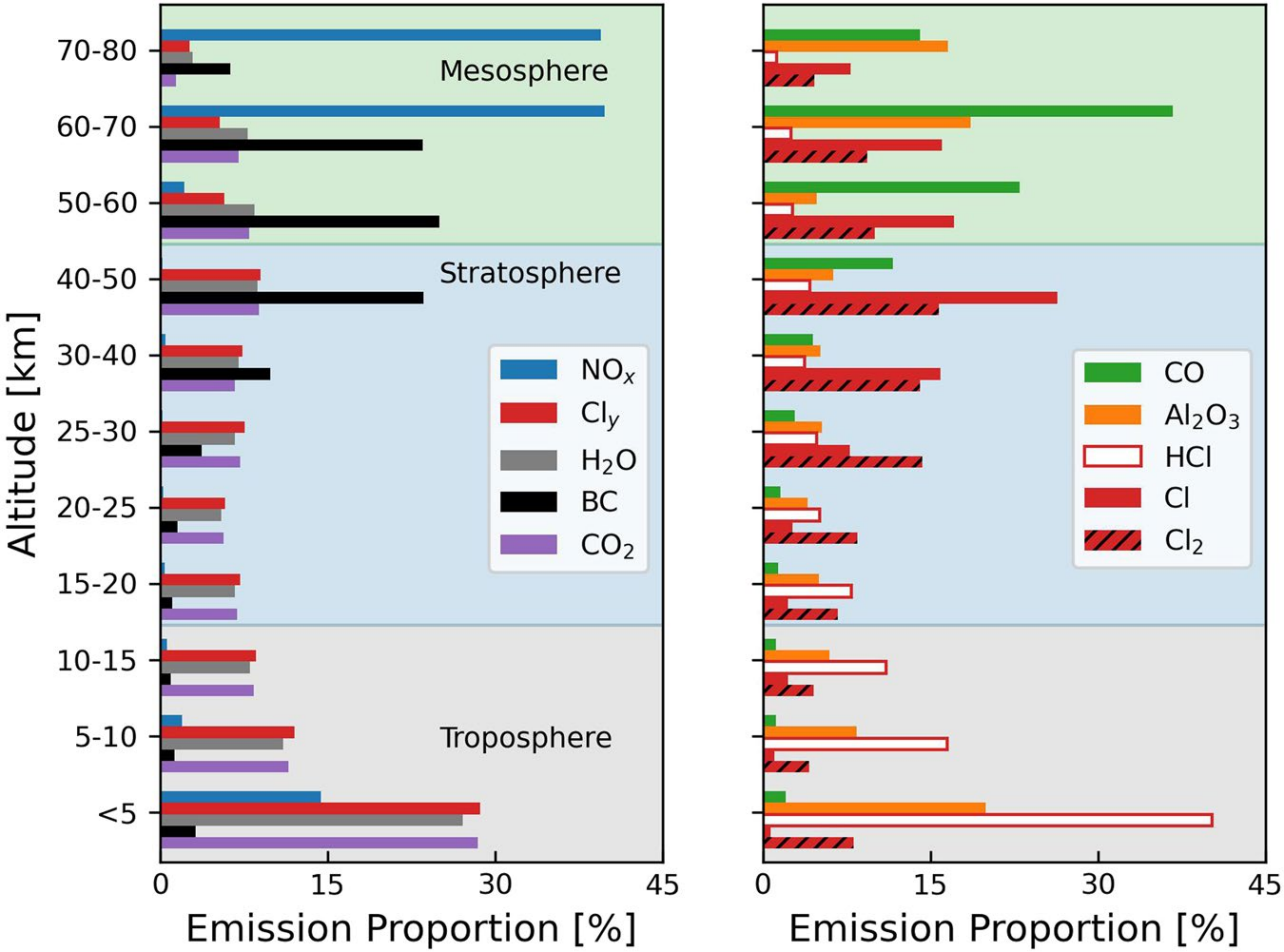
Vertical distribution of air pollutant and CO_2_ emissions from rocket launches and object re-entries in 2022. Emissions totals are in Fig. 6.

**Fig. 6:**
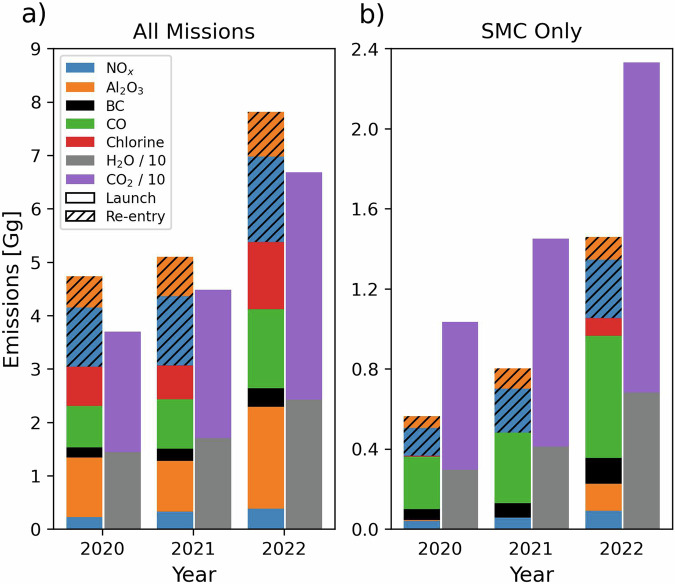
Annual rocket launch and re-entry air pollutant and CO_2_ emissions in 2020–2022. Panels are for all launches and object re-entries (**a**) and for SMC missions only (**b**). H_2_O and CO_2_ emissions are divided by 10 to fit within the plot range.

Figure 5 shows the vertical distribution of air pollutant and CO_2_ emissions from rocket launches and object re-entries in 2022. The distribution is similar in 2020 and 2021. For all byproducts except HCl, most (51–96%) emissions occur above the tropopause (>15 km). As a result of afterburning, most (>78%) CO and BC emissions occur above 40 km. Total emissions in each year, shown in Fig. 6a, increased from 2020 to 2022 by 42% for re-entry Al_2_O_3_ to 91% for CO. The large increase in CO is due to an increase in Falcon 9 kerosene rocket launches from 25 in 2020 to 61 in 2022. Figure 6b shows emissions attributable to SMC missions that have doubled from 2020 to 2022 for re-entry Al_2_O_3_ and increased 23-fold for launch Al_2_O_3_ and Cl_y_. SMCs make the largest proportional contribution to carbon-based emissions, due to dominance of kerosene-fuelled rockets. By 2022, carbon-based emissions from SMCs are 37% of total BC emissions, 41% for CO, and 39% for CO_2_. Only 16 of the 86 SMC missions used non-Falcon rockets, though the propellant for many of these is also kerosene. Non-Falcon SMC mission rockets include Kuaizhou-1 (solid/hypergolic), Soyuz-2-1B Fregat series (kerosene/hypergolic), CZ-2C (hypergolic), Electron (kerosene/hypergolic), and GSLV Mk III (solid/hypergolic/hydrogen). As a result, SMCs are only 7% of total emissions of Cl_y_ and Al_2_O_3_ by 2022.

## Data Records

The dataset and files detailed in Tables 2–5 are available via the UCL Data Repository^76^. This section describes the contents of the data. The compiled and cross-checked data of rocket launch and re-entry object activities for 2020–2022 are provided in three NetCDF formatted files. Specific variables of these files are described in Table 2 for each launch (file named launch_activity_data_2020–2022.nc), Table 3 for each type of rocket (rocket_attributes_2020–2022.nc), and Table 4 for all re-entries (reentry_activity_data_2020–2022.nc).

**Table 2:** Variables in the launch_activity_data_2020–2022.nc file detailing each 2020–2022 launch.

Variable	Type	Description
COSPAR_ID	object	Unique Committee on Space Research (COSPAR) launch ID, in the form YYYY-NNN for successful launches, where 2020-001 is the first successful launch in 2020. Failed launches are in the form YYYY-FNN.
Time (UTC)	float64	Time of liftoff in Coordinated Universal Time.
Date	object	The date of the launch in the form YYYYMMDD.
Longitude/Latitude^a^	float64	Location of the launch in decimal degrees.
Rocket_Name	object	Launch vehicle name. Used to identify corresponding rocket information in the rocket_attributes_2020–2022.nc dataset.
DISCOSweb_Rocket_ID	object	Launch vehicle ID from DISCOSweb. Required to generate rocket_attributes_2020–2022.nc file.
Megaconstellation_Flag	bool	Megaconstellation True/False identifier flag. True for launches containing satellite megaconstellation payloads (rideshare or exclusive).

^a^“/” indicates each is a separate entry in the file.

**Table 3:** Variables in the rocket_attributes_2020–2022.nc file detailing each type of rocket launched in 2020–2022.

Variable	Data Type	Description
Rocket_Name^a^	object	Rocket name. Used to identify corresponding launches in the launch_activity_data_2020–2022.nc dataset.
Booster_No	object	Number of boosters, ranging from 0 to 6.
<Stage>_PropMass^b^	float64	Propellant mass of each stage in kg. For boosters, this is the sum of all boosters. 0 if stage is absent.
<Stage>_Fuel_Type^b^	object	Propellant category for each stage. Either Kerosene, Hydrogen, Solid, Hypergolic, or Methane. Empty if stage is absent.
<Stage>_StageMass^b^	float64	Dry mass of each stage in kg, used to cross-check re-entry mass of rocket stages. For boosters, this is the sum of all boosters. 0 if stage is absent.
Fairing_Mass	float64	Dry mass of launch vehicle fairing in kg (combined total of both halves), used to cross-check fairing re-entry mass.
Proxy_Rocket	object	Name of rocket used as proxy for rockets missing data. Empty if no approximation needed.

^a^Atlas V rocket upgrades are distinguished with the name “Atlas V XXX” before upgrade, “Atlas V XXX v2020” after boosters upgraded in 2020, and “Atlas V XXX v2021” after upper stages upgraded in 2021, where XXX is the rocket model number e.g. 501 (4 models for the original rocket, 6 for v2020, and 1 for v2021). Each model differs in stage and propellant mass. ^b^The data variable name <Stage> is either Booster, Stage1, Stage2, Stage3 or Stage4.

**Table 4:** Variables in the reentry_activity_data_2020–2022.nc file detailing each object re-entering in 2020–2022.

Variable	Type	Description
COSPAR_ID	object	COSPAR object ID, in the form YYYY-NNNX, where X is the X^th^ object associated with that launch, e.g. 2020-001 A is the first object from launch 2020-001. Non-orbital objects are not assigned a COSPAR object ID, and so we use the COSPAR launch ID.
Object_Name	object	The name of the returning object as given in the GCAT, DISCOSweb and Aerospace Corp. databases.
Category	object	Letter assigned to object category. “Bn” for boosters, where n is the booster number from 1 up to 6, “Sn” for stage numbers where n is stage number from 1 up to 4, “P” for payload, “F” for fairing, and “C” for component (e.g. deployment rails, smaller objects).
Time (UTC)	float64	The time of re-entry in Coordinated Universal Time.
Date	object	The date of the launch in the form YYYYMMDD.
Longitude/Latitude^a^	float64	The location of the re-entry in decimal degrees.
Ablatable_Mass^b^	float64	The dry mass of the re-entering object in kg.
Ablation_Degree	float64	The percentage of Ablatable_Mass ablated on re-entry.
Percent_Aluminium	float64	The percentage of Ablatable_Mass present as aluminium.
Other_Mass^b^	float64	Additional mass (propellant and cargo) of re-entering objects in kg that does not ablate but does contribute to re-entry NO_x_ emissions.
Megaconstellation_Flag	bool	A megaconstellation True/False identifier flag. True for objects with a COSPAR ID corresponding to launches containing satellite megaconstellation payloads (rideshare or exclusive).
Location_Constraint	int64	A diagnostic flag specifying the location constraint used to map re-entries. Integer values from 1 to 7 defined by location constraint: (1) Latitude and longitude, (2) Launch site or location, (3) Political region (e.g. country/state), (4) Physical region (e.g. ocean/continent), (5) Falcon landing/recovery, (6) Inclination-bounded random, (7) Unbounded random.
Apogee	float64	The maximum altitude of the object, giving users the autonomy to exclude re-entering objects below an apogee other than the 50 km threshold used here.

^a^“/” indicates each is a separate entry in the file. ^b^Sum of Ablatable_mass and Other_mass yields total re-entry mass.

**Table 5:** Variables in each daily byproduct_emis_<mission_type>_<horiz_res>_<vert_res>_YYYYMMDD.nc4 file^a^.

Variable	Type	Description
time	int64	Time coordinate in hours since YYYYMMDD 00:00:00 UTC, where YYYYMMDD is the date in the filename.
lon	float32	Longitude of grid box centre in degrees east.
lat	float32	Latitude of the grid box centre in degrees north.
lev	int64	Grid box level number.
launch_nox_thermal	float32	Indirect launch emissions of NO_x_ in kg m^−2^ s^−1^.
launch_nox_fuel	float32	Direct launch emissions of NO_x_ from propellant combustion in kg m^−2^ s^−1^.
launch_<byproduct>^b^	float32	Launch byproduct emissions in kg m^−2^ s^−1^.
reentry_<byproduct>^c^	float32	Re-entry byproduct emissions in kg m^−2^ s^−1^.

^a^Provided in Cooperative Ocean Atmosphere Research Data Service (COARDS) compliant format for <mission_type> “nonsmc”, “smc”, or “all”, <horiz_res> “4 × 5” or “2 × 25”, and <vert_res> “47” or “72”. ^b^Launch <byproduct> is CO_2_ and all air pollutants (H_2_O, BC, Al_2_O_3_, HCl, Cl, Cl_2_, CO) except NO_x_. ^c^Re-entry <byproduct> is NO_x_ and Al_2_O_3_.

Table 5 describes the variables in each daily file containing the vertically and horizontally gridded hourly air pollutant and CO_2_ emissions. Multiple sets of daily files are provided for all missions, SMC missions only, and non-SMC missions. These file sets are the emissions at a range of horizontal and vertical resolutions. These include global model representative coarse horizontal (4° × 5°; latitude × longitude) and vertical (47 layers to 0.01 hPa) resolutions and finer horizontal (2° × 2.5°) and vertical (72 layers to 0.01 hPa) resolutions, totalling 12 file sets. To minimize file sizes, each daily file is trimmed to the latitude and longitude range containing the rocket launch and re-entry emissions.

## Technical Validation

Beyond rigorous cross-checking of primary data against independent, reliable sources (Fig. 1), we also validate the vertical distribution of propellant mass burned using publicly available launch livestreams and our emissions against previously published values. Further validation is not feasible, as there is a lack of experimental data and real-world observations of byproducts from modern rocket launches and object re-entries.

### Evaluation of propellant consumption profiles

We assess our approach of vertically distributing propellant consumption with livestreams of launches in 2020. Livestreams of 32 launches of mostly SpaceX Falcon 9 rockets, available on YouTube or launch provider websites, include real-time data of launch altitude and time. The data we gather from these livestreams include the time elapsed in 5 km altitude intervals, and the altitude and time of BECO, MECO, SEI, and SECO. Though SECO usually occurs well above the altitude limit of our inventory, it is necessary to estimate second stage propellant burned in the target altitude range. We use the total time from rocket stage engine ignition to cut-off, propellant mass consumed by each stage from our launch database, and the 5-km time interval data to calculate the mass of propellant burned in each 5-km bin up to 80 km. This approach assumes that each rocket stage burn rate is constant with altitude and so ignores throttling of rocket stages. Engine throttling is a ubiquitous technique to vary the propellant burn rate of boosters and main stages by reducing thrust during maximum dynamic pressure to minimise structural damage to the rocket^25,67,77^. Maximum dynamic pressure typically occurs in the upper troposphere at ~10 km^35,48,78,79^, so ignoring throttling likely overestimates propellant consumption in the 5–15 km bins and slightly underestimates propellant consumption in all other bins covering the altitude range of the boosters and main stages.

**Fig. 7:**
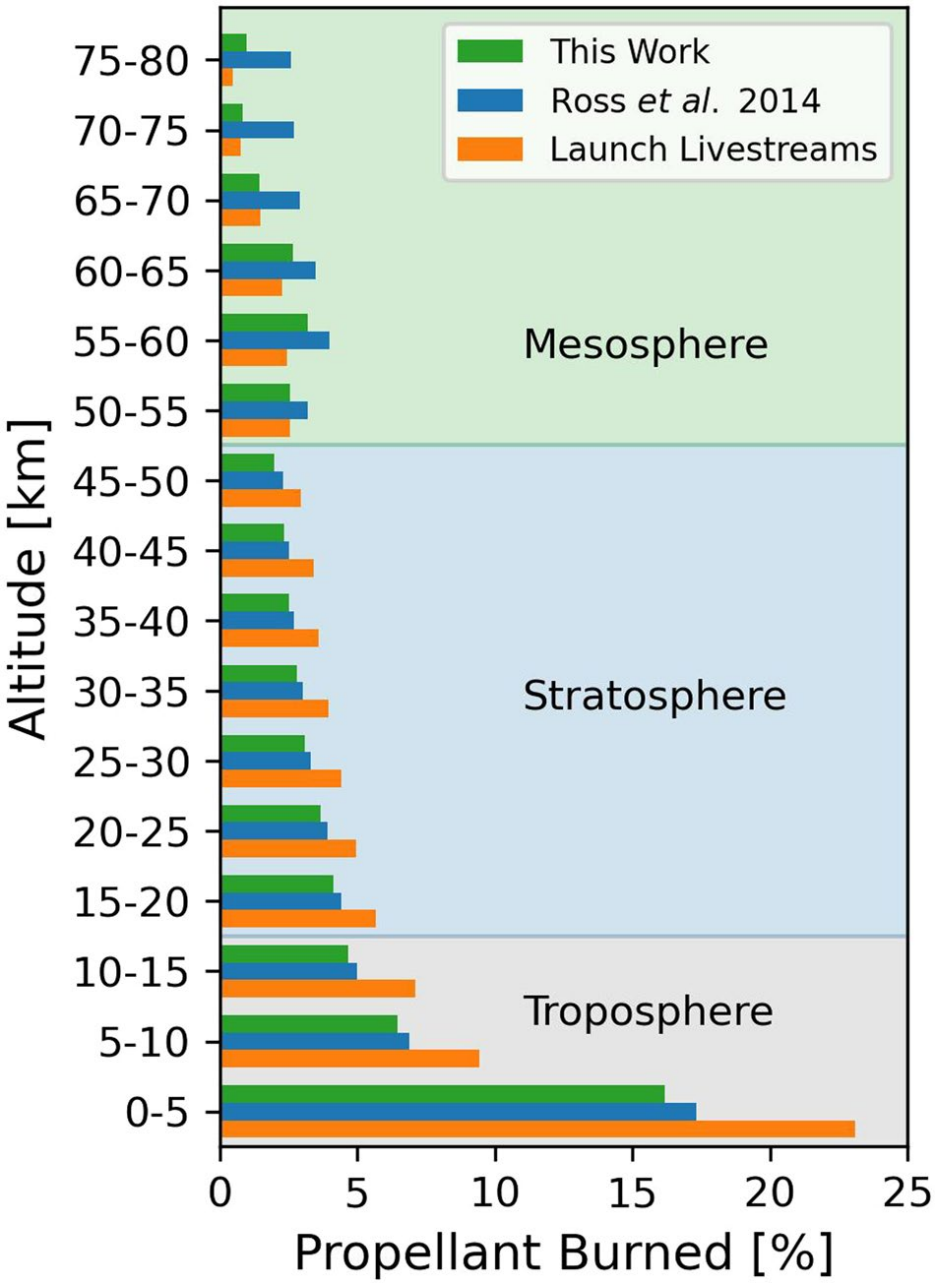
Evaluation of vertical profiles of propellant consumption. Profiles are propellant burned in 5-km bins up to 80 km and include those used in this work (green), reported in the literature^20^ (blue), and obtained from launch livestreams (orange) (see text for details). Shading distinguishes the troposphere (grey), stratosphere (blue), and mesosphere (green).

Figure 7 compares our propellant consumption vertical profiles to the literature profile^20^ on which our vertical profiles are based and to vertical profiles obtained from launch livestream data. Our vertical profiles and the launch livestream profiles are averaged over the same 32 rocket launches with launch livestream data in 2020 to ensure a consistent comparison. The literature profile should be broadly similar to these too, as most launch livestreams are for Falcon 9 2-stage rockets and the literature profile is derived for a rocket without boosters. Our methodology places 27% of emissions into the troposphere (grey shading in Fig. 7), similar to 29% using the literature distribution. The launch livestream tropospheric contribution is greater than both the other profiles at 40%, due to omission of engine throttling in the troposphere. Proportions for the stratosphere (blue shading) are similar between each profile, with 20% for this work, 22% for the literature distribution, and 29% for the launch livestreams. Our propellant profile is most consistent with the launch livestream values in the mesosphere (green shading) at 12% for this work and 10% for the launch livestreams, compared to 19% for the literature distribution. The proportion burned above 80 km is then 41% for our profile, 30% for the literature profile, and 21% for the livestreams. We use this validation to estimate the uncertainty in our propellant consumption profile and find that our profile is 37% lower than the launch livestream profile in the troposphere, 35% lower in the stratosphere, and 9% higher in the mesosphere.

### Evaluation of air pollutant and CO_2_ emissions

It is not feasible to directly compare our launch emissions to past studies, as there are no other launch emissions estimates for 2020–2022 and launch rates have grown by 38% a^−1^ from 2020 to 2022, compared to 5.6% a^−1^ from 2003 to 2019^9^. Given this rapid increase in launch rates after 2019, we calculate per-launch emissions of air pollutant and CO_2_ for our 2020 emissions and for literature values from the Ryan *et al*.^9^ study of 2019 air pollutant emissions, the Desain and Brady^24^ study of 2013 air pollutant and CO_2_ emissions, and the Pradon *et al*.^25^ study of 2018 H_2_O emissions. We calculate similar (just 2% more) per-launch emissions of NO_x_ to Ryan *et al*., and 59% more NO_x_ than Desain and Brady. The latter study calculated NO_x_ emissions by extrapolating values from simulated NO_x_ mass flow experiments^80^. Our approach of comparing across emission years assumes a similar rocket fleet and propellant mix in each year. Solid propellant consumption has declined over time, decreasing Al_2_O_3_ and Cl_y_ emissions. A third of rocket launches in 2013 used solid propellant and the proportion of propellant mass declined from 14% in 2019^9^ to 10% in 2020. As a result, our per-launch emissions of Cl_y_ are 26–41% less than the other studies, and our Al_2_O_3_ emissions are 39% less than Desain and Brady and 37% less than Ryan *et al*., though the latter also includes differences in EIs. Ryan *et al*. did not account for afterburning effects, leading to 66% less BC and 26% more H_2_O per-launch emissions in our inventory than in Ryan *et al*. Desain and Brady only appear to account for afterburning effects of NO_x_ and BC, not CO_2_ or H_2_O, and so we calculate 53% more CO_2_ and 25% more H_2_O per-launch emissions in our inventory than in Desain and Brady. We calculate 15% less H_2_O emissions than Pradon *et al*., as their emissions estimate extends to 100 km.

We also compare our object re-entry mass and Al_2_O_3_ and NO_x_ emissions to literature studies for 2019 (Ryan *et al*.^9^, Schulz and Glassmeier^10^), 2020–2021 (Jain 2023^28^), and 2022 (Ferreira *et al*.^27^). We calculate greater re-entry mass and more Al_2_O_3_ emissions in 2020 than the literature values for 2019. This increase is in part due to an increase in objects re-entering^7^, but is mostly due to the filtering used when constructing the re-entry databases. We include suborbital objects and objects with apogee >50 km. Ryan *et al*. only included orbital, geolocated re-entries^9^ that in our database are ~64% of total re-entry mass. Our re-entry mass influx for 2020 exceeds the Ryan *et al*.^9^ estimate by 0.7 Gg. Schulz and Glassmeier only accounted for suborbital objects with a reported or inferred velocity ≥3.8 km s^−1^, resulting in an annual mass influx of 0.89 Gg (28% of this study) and Al emissions of 0.21 Gg (36% of this study). The Jain study estimated emissions by excluding suborbital objects, omitting nearly half (46%) of all re-entries^28^, yielding only 0.04 Gg Al_2_O_3_ emissions in 2020 compared to our 0.59 Gg. Ferreira used molecular dynamics simulations to model ablation emissions of Al_2_O_3_ from LEO payloads only. They report 17 tonnes of Al_2_O_3_ emissions in 2022 from a re-entry mass influx of 146 tonnes. Our values for the equivalent re-entering orbital payloads in 2022 are 182 tonnes re-entry mass and 32 tonnes Al_2_O_3_. We estimate more Al_2_O_3_ than Ferreira, as they convert 32% of re-entry Al to Al_2_O_3_, whereas we convert all Al to Al_2_O_3_. We calculate significantly less re-entry NO_x_ in 2020 (1.1 Gg) than in 2019 reported by Ryan *et al*. (1.9 Gg), as our re-entry emission index for indirect thermal NO_x_ is 40% compared to 100% in Ryan *et al*.

Overall, the launch and re-entry emissions we calculate in comparison to past studies is within the range of uncertainty expected for these estimates, given the assumptions required in the absence of constraints from real-world measurements.

## Usage Notes

We provide air pollutant and CO_2_ emissions with an hourly timestep, horizontal resolutions of 4° × 5° and 2° × 2.5° (latitude × longitude) and vertical resolutions of 47 and 72 layers up to 80 km (0.01 hPa). The final emissions are in NetCDF files for use with atmospheric chemical transport or Earth system models to determine atmospheric impacts of the emissions. The emissions can be directly input to the GEOS-Chem model or models that include emissions processing packages that interpolate emissions to the horizontal model resolution.

We also provide a Python v3.9 script (calculate_gridded_emissions_for_2020–2022.py) and an input file (define_grid_resolution_timestep.txt) for users to recalculate the emissions to other model timesteps, vertical and horizontal resolutions, and vertical extents (limited to 100 km), or to use different primary emission indices (set in primary_emission_indices.csv). An hourly time resolution is used to match the time resolution of the emissions processing package in the GEOS-Chem model, but users can also reprocess the emissions to be at the launch and re-entry times and to occur over timescales typical of these processes (2–4 min for launches; <2 min for re-entries). All launch and re-entry emissions are injected into a fixed vertical column based on the latitude and longitude provided in the launch and re-entry databases. Rockets deviate from the launch site by <350 km from above an altitude of ~120 km^81^ and anthropogenic re-entering objects travel ~300 km horizontally during re-entry ablation^8^. Given this and the uncertainties in geolocating re-entering objects, our emissions inventory is most suitable for horizontal resolutions of 2° (~200 km) and coarser. Re-entry locations include a random component. The data provided match the spatial distribution in Fig. 3. Recomputing the inventory of re-entering object mass using the compile_reentry_data.py script will redistribute the randomly gridded re-entering objects (blue crosses in Fig. 3a).

## Data Availability

The Python scripts (Python v3.9) used to compile the launch and rocket information from DISCOSweb (compile_rocket_launch_data.py) and cross-check the launch and rocket data against primary and ancillary data sources (update_rocket_launch_data.py) (Fig. 1) are publicly available via Zenodo^82^. We also provide scripts to compile and cross-check the re-entering object information (compile_reentry_data.py), and to generate and grid the launch and re-entry emissions (calculate_gridded_emissions_for_2020–2022.py). Also included are input files (define_grid_resolution_timestep.txt, primary_emission_indices.csv, and launch_event_altitudes.csv) and files listing Python modules (module_list.txt) and databases (database_list.txt) required to run the scripts.
